# Neuromesodermal Lineage Contribution to CNS Development in Invertebrate and Vertebrate Chordates

**DOI:** 10.3390/genes12040592

**Published:** 2021-04-17

**Authors:** Clare Hudson, Hitoyoshi Yasuo

**Affiliations:** Laboratoire de Biologie du Développement de Villefranche-sur-mer (LBDV), Institut de la Mer de Villefranche, Sorbonne Université, CNRS, 06230 Villefranche-sur-mer, France; yasuo@obs-vlfr.fr

**Keywords:** ascidian, CNS, neural induction, anterior-posterior patterning, NMps, neuromesoderm precursor, axial precursor, spinal cord

## Abstract

Ascidians are invertebrate chordates and the closest living relative to vertebrates. In ascidian embryos a large part of the central nervous system arises from cells associated with mesoderm rather than ectoderm lineages. This seems at odds with the traditional view of vertebrate nervous system development which was thought to be induced from ectoderm cells, initially with anterior character and later transformed by posteriorizing signals, to generate the entire anterior-posterior axis of the central nervous system. Recent advances in vertebrate developmental biology, however, show that much of the posterior central nervous system, or spinal cord, in fact arises from cells that share a common origin with mesoderm. This indicates a conserved role for bi-potential neuromesoderm precursors in chordate CNS formation. However, the boundary between neural tissue arising from these distinct neural lineages does not appear to be fixed, which leads to the notion that anterior-posterior patterning and neural fate formation can evolve independently.

## 1. Introduction

Ascidians are marine invertebrate chordates that are the closest living relatives to the vertebrates [1]. This review is concerned with the tadpole larvae of solitary species, predominantly *Halocynthia roretzi* and *Ciona* sp. (*Ciona intestinalis*, *Ciona robusta* and *Ciona savigni*, which will be discussed as “*Ciona*”). *Halocynthia* and *Ciona* belong to different orders of ascidians, but they develop in an almost identical manner in terms of cell lineages and embryo anatomy and morphology even if not necessarily via conserved developmental molecular mechanisms [2,3,4,5,6,7,8,9,10]. At larval stages of development, ascidians possess a notochord and a dorsal neural tube, both synapomorphies of chordates [11]. Ascidian embryos develop with a fixed cell lineage [5]. Most cells are fate-restricted at the onset of gastrulation, when the embryo consists of 112 cells. The larva is composed of in the region of 3000 cells [12]. No stem cells are involved in generating the larval body and the tail elongates by a process of convergence and extension [13,14].

A long-held traditional view of vertebrate CNS formation is that neural tissue is induced from the ectoderm germ layer in a process called “neural induction”, cells choosing between neural and epidermis. This neural induction step induces neural fate with anterior character, part of which is then “transformed” into posterior fates by posteriorizing signals (reviewed in [15,16]). This seemed at odds with the observation in ascidian larvae that the anterior and posterior CNS arise from distinct hemispheres of the early embryo, with animal hemisphere-derived anterior neural tissue arising from binary fate choices with epidermis and the vegetal hemisphere-derived posterior CNS arising from binary fate choices with mesoderm. However, in vertebrates, while some posterior neural tissue does form via a process known as “posteriorization”, it has become clear that the posterior-most CNS originates from a distinct ontology to the anterior CNS and is associated with mesoderm fates [15,17,18,19]. These recent advances may bring the ontologies of the ascidian and vertebrate CNSs closer together than was once believed. Throughout this review we will adopt the following definitions of bi-fated and bi-potential [20]. Bi-fated precursors are those that generate two tissue types (neural and mesoderm, for example), whereas mono-fated implies lineage restriction of a cell to a specific tissue type. This definition does not necessarily imply any type of commitment of cells to a particular fate [21]. We refer to bi-potential precursors as those that can adopt one of two alternative fates based on the signals they receive; i.e., they are competent to form the two cell types, depending upon their environment. This does not rule out the possibility that the local embryonic environment may impose mono-fated development from precursors that are nonetheless bi-potential, with bi-potentiality only being revealed by experimental manipulation. In this review, we provide a detailed description of the embryonic origin of the ascidian larval neuromesoderm-derived CNS and discuss to what extent this is similar to the situation in vertebrates, with particular focus on mice, chick and zebrafish as vertebrate examples.

## 2. The Ascidian Larval CNS

The ascidian larval neural tube forms through the rolling up of the neural plate, similarly to primary neurulation in vertebrates [14,22,23]. The ascidian larval CNS has been extensively studied in terms of its lineage, molecular mechanisms of development, gene expression patterns and cell types [23,24,25,26,27]. More recently, a larval nervous system connectome has been revealed [28] and collections of single cell transcriptomics generated [29,30,31,32,33]. Emerging studies are now aiming to annotate each neuron identified in the connectome with the gene expression patterns, including neurotransmitter types, described from in situ hybridization/immunostaining analysis or single cell transcriptomics datasets, as well as to the cell lineage history and ultimately to neuronal function and larval behavior [29,34,35,36,37,38,39].

**Figure 1:**
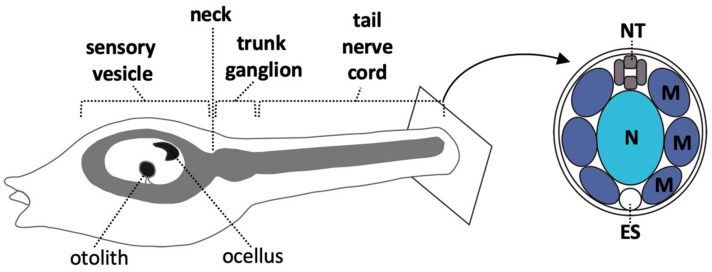
Morphological features of the larval CNS. Schematic drawing of the larval CNS, enlarged within the larval shape outline for illustration purposes. Right is a cross-section though the tail showing the chordate organization of axial notochord (N, light blue), lateral muscles (M, dark blue) and the neural tube with a four-cell configuration (NT, dark grey). ES = endodermal strand.

At larvae stages, the ascidian CNS has distinct morphological units which are, from anterior to posterior, the sensory vesicle, a narrow ‘neck’ region, a trunk ganglion, and a tail ‘nerve cord’ (Figure 1) [14]. In the literature, the posterior part of the sensory vesicle is sometimes referred to as the posterior brain [23,24,40] or brain stem [8]. The sensory vesicle contains two pigmented sense organs, an otolith, anchored in the ventral wall and used for gravity perception, and an ocellus, required for photo-reception [24,25,41,42,43]. The trunk ganglion [44] is also referred to as the visceral ganglion [24,45] or motor ganglion, since it contains the motor neurons that innervate the tail muscle [23,28,46,47,48]. Here we use the morphology-based term, ‘trunk ganglion’. The nerve cord is the posterior-most part of the CNS; a tube consisting of only 4 cells in cross section, one dorsal, one ventral and two lateral, though nonetheless, it has recently been shown to harbor neurons [28,47,49,50]. The ascidian larvae CNS is made up of approximately 330 cells of which at least 177 are neurons, located in the sensory vesicle (143), trunk ganglion (25) and tail nerve cord (up to approximately 20) [27,28,47,49,51]. Transcriptomic analysis has identified 41 distinct neural subtypes in larva [29]. For a description of some of these neural cell types, see recent reviews [24,25,27].

Aside from the neurons, the non-neuronal ependymal cells of the CNS (apart from those residing in the tail nerve cord) form an important stem cell-like source that will later give rise to the post-metamorphosis adult nervous system [40,44]. Some larval neurons also persist after metamorphosis, although many others are lost during the process [40].

## 3. Anterior-Posterior Regionalization of the Chordate Neural Tube

**Figure 2:**
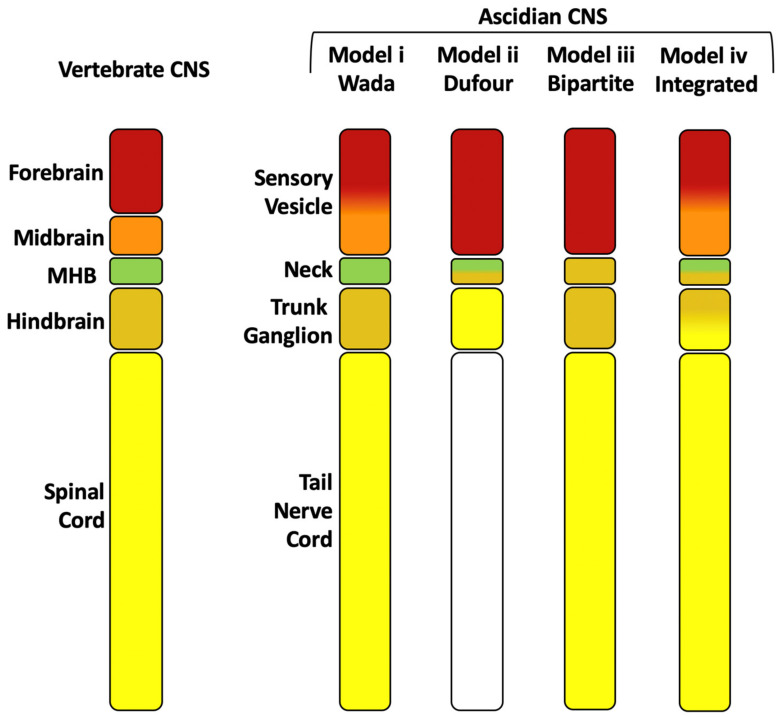
CNS organization along the anterior-posterior axis in ascidians and vertebrates. Left is a schematic drawing of the vertebrate CNS compartments indicated as boxes, with forebrain colored in red, midbrain in orange, mid-hindbrain (MHB) in green, hindbrain in tan and spinal cord in yellow. Right are four models of the ascidian CNS with the different morphological compartments (sensory vesicle, neck, trunk ganglion and tail nerve cord) indicated as boxes. Colors indicate equivalence to vertebrate CNS compartments in four different models. White indicates no (or uncertain) homology. Models are based on information in [63]-Model i; [44]-Model ii; [57]-Model iii; with Model iv showing an integrated summary of previous and recent data (see text for details).

Regional comparisons between ascidian larval and vertebrate CNSs are complicated by a significant simplification of the ascidian CNS that most likely took place in the tunicate lineage [23,52,53,54,55], whereas the vertebrate CNS has evolved more complex structures [55,56]. Nonetheless, comparisons of gene expression profiles, neural function and connectivity along the anterior-posterior axis of the CNS between ascidians and vertebrates can go some way towards providing equivalence between the different domains and have led to several hypothesis (Figure 2) [25,26,44,54,57,58,59,60,61,62,63,64]. At the anterior, the *Otx* positive domain, or sensory vesicle, likely corresponds to the vertebrate forebrain. Within the *Otx* positive domain, the presence or absence of a midbrain has been the subject of much debate (Figure 2, models ii and iii have no midbrain equivalent) [26,54,57,62]. However, recent evidence on the function of the posterior sensory vesicle as a sensory processing and integration center, as well as gene expression patterns and neuronal circuitry has suggested that part of the posterior sensory vesicle could be homologous to the vertebrate midbrain [34] (Figure 2, model iv). Between the sensory vesicle and trunk ganglion, the small intervening neck region (not morphologically recognizable in *Halocynthia* but identifiable by gene expression) of only 6 cells [51], is difficult to assign to a specific vertebrate counterpart, which is further confounded by dynamic gene expression in this region [58]. At late tailbud stages, however, the neck region is neither *Otx* nor *HOX* positive [58,64]. This small region may therefore correspond to the mid-hindbrain boundary (MHB; Figure 2 models i, ii) although the presence of a MHB in tunicates is also debated (Figure 2, model iii) and if present, it does not appear to have an organizer function as it does in vertebrates [26,54,57,58,59,63,64]. There is also evidence that the neck region may, in part, correspond to the hindbrain, since some cells labelled by *Phox*, located in this region, give rise to the visceral motoneurons of the adult, a neuronal cell type that arises in the hindbrain of vertebrates (Figure 2 model ii) [44].

The trunk ganglion contains the motor neurons that control swimming behavior [24,47,65,66] and expresses *HOX* genes, and has been compared to either the spinal cord (Figure 2 model ii) [44,50,65] or hindbrain (Figure 2, model i, iii) [57,62,63]. A specific gene expression pattern of transcription factors along the anterior-posterior axis of 5 pairs of cholinergic neurons (descending decussating neuron (ddN), interneurons and motoneurons) of the trunk ganglion, which is conserved among different ascidian species, has been compared to a similar order of gene expression along the dorsal-ventral axis of the vertebrate spinal cord [7,67]. Together with the ascending commissural inhibitory interneurons (ACINs) in the anterior nerve cord, the cholinergic motoneurons of the trunk ganglion are comparable to the vertebrate spinal cord central pattern generators that generate rhythmic swimming behavior [65]. However, the most anterior of the trunk ganglion cholinergic neurons, a pair of descending decussating neurons (ddNs), have been likened to the Mauthner cells of tailed vertebrates, neurons which arise in the hindbrain and are involved in the startle response network [68]. At midgastrula stages, *FGF8/17/18* is expressed in lateral trunk ganglion precursors just posterior to the neck precursors and it is required for the specification of the neck region itself, which otherwise adopts a posterior sensory vesicle-like fate [26]. This result could be interpreted as a posterior shift in MHB FGF-signaling in the ascidian larval CNS relative to the vertebrate CNS [26,58]. Alternatively, the *FGF8/17/18*-positive region could be similar to the hindbrain FGF-signaling activity located at rhombomere 4 of vertebrate embryos [57,69,70,71]. We conclude that the trunk ganglion may have homology to both the hindbrain, at least at its anterior end (Mauthner-like cells), and the spinal cord (somatic motoneurons of the central pattern generator) (Figure 2, model iv).

The tail nerve cord is likely to be homologous to the vertebrate spinal cord [57,64] (Figure 2 model i, iii, iv). While it was originally believed that the tail nerve cord was devoid of neurons and was thus removed from any comparative analysis in model ii [44], we now know that neurons are present in the tail nerve cord [28,36,47,65]. The anterior part of the tail nerve cord contains motoneurons (4 cells) and interneurons (2 cells) [27] and the ACINs (usually 3–4 cells) [28,36,47,65,68], that are involved swimming behaviors [47,65,68]. Other cholinergic mid-tail motoneurons or ‘planate neurons’ are located along the tail nerve cord [27,47,68] of which 12-15 cells have been observed [47].

We propose a model (Figure 2, model iv) whereby the sensory vesicle represents the vertebrate *Otx* positive ‘forebrain’ and ‘midbrain’, with the midbrain corresponding to part of the posterior sensory vesicle [34], the intervening neck region, which is *Otx*- and *HOX*-negative at some stages, representing the ‘mid-hindbrain’ and ‘hindbrain’, the trunk ganglion representing a ‘hindbrain-spinal cord’ region and the remaining tail nerve cord, with its neurons, corresponding to the rest of the ‘spinal cord’. This model (iv) fits a tripartite organization, with an *Otx* positive anterior domain, a posterior *HOX* positive domain with an *Otx/HOX* negative MHB domain in between [25,44,58,61,63,64]. However, it has also been suggested that the ascidian CNS could instead be considered bipartite, with only a forebrain and a hindbrain/spinal cord, and lacking a potential MHB domain (Figure 2 model iii) [54,57,58]. It is likely that the debate will continue and boundaries will be refined as more data on neural function and gene expression is acquired.

## 4. A Large Part of the Ascidian CNS Is Derived from Bi-Fated Neuromesodermal Precursors

**Figure 3:**
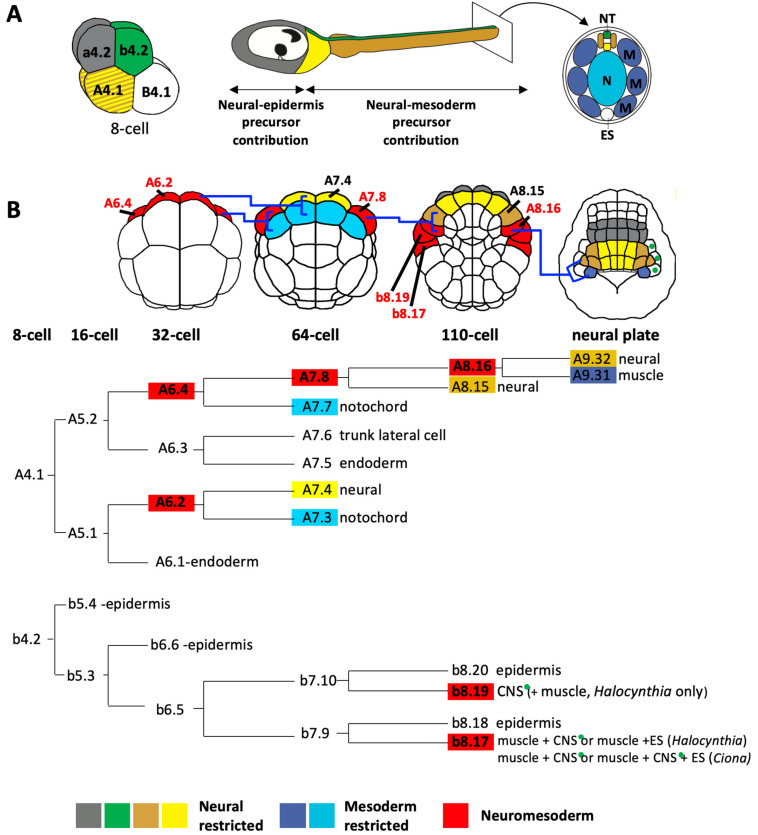
Lineage contributions to the larval CNS. (**A**) 8-cell stage embryo in lateral view (left) showing the four founder lineages, a4.2 (grey) and b4.2 (green) of the animal hemisphere and A4.1 (tan/yellow) and B4.1 (white) of the vegetal hemisphere. Middle shows a schematic drawing of the larval CNS with the contributions from the a4.2 (grey), b4.2 (green) and A4.1 (tan and yellow) shown and summarizing the main contributions from neural-epidermis precursors and neural-mesoderm precursors. Right is a cross-section though the tail showing the chordate organization of axial notochord (N, light blue), lateral muscles (M, dark blue) and the neural tube (NT, green, yellow and tan). For NT, contributions of founder lineages are indicated with colors whereas notochord derives from A-lineage (mostly) and B-lineage and muscle derives from B-(mostly), A- and b-lineage. ES-endoderm strand. (**B**) Schematic drawings of embryos in vegetal pole view and cell lineages of one A4.1 and one b4.2 showing the bi-fated neuromesoderm precursors in red. Other colors indicate fate-restricted lineages following the same color code as (A) The green dots indicate b-line CNS precursors, the fate segregation of which is not fully resolved.

Ascidian embryos develop with a fixed cell cleavage pattern and lineage, allowing the exact lineage relationships between different cell types to be known as long as the cells can be traced and identified [3,8,13,26,37,38,50,58]. The embryos are bilaterally symmetrical so each cell name refers to a pair of cells [72]. At the 8-cell stage of development, the embryo divides along the animal-vegetal axis to generate the four founder lineages. The four animal cells, the a4.2 and b4.2 cell pairs, generate the a- and b-lineages and the four vegetal cells, the A4.1 and B4.1 cell pairs, generate the A- and B-lineages (Figure 3A). a- and b-lineages give rise to predominantly ectoderm: all of the epidermis and the part of the CNS [8,14,45,73]. The vegetal lineages (A- and B-) will generate most of the endoderm and mesoderm but, importantly, a large part of the posterior CNS is also derived from the A-lineages (Figure 3A) [8,14,45,73]. 

The following description of the neural lineages is based on a number of published studies [8,13,45,74,75]. The a-lineages will generate the anterior part of the sensory vesicle (Figure 3A, grey). The process of neural induction in a- (and b-) line cells begins at the 32-cell stage with induction of the *Otx* gene in two pairs of neural lineage precursors, following a cell fate choice between neural and epidermal tissue [76,77,78]. At the early gastrula stage the a-lineage neural precursors segregate further into anterior sensory vesicle precursors and, more anteriorly, a specialized region of anterior ectoderm that has been likened to a proto-placodal territory that generates the palps (a sensory adhesive organ) as well as certain epidermal sensory neurons [30,74,79,80]. Manipulating the inducing signal or transcription factor expression during these cell fate decisions suggests that they operate in a binary mode: epidermal vs neural, CNS vs sensory placode [78,81,82,83]. Interestingly, promotion of neural fates in a-lineages involves similar factors (the transcription factors Foxa.a and Zic.r.b and FGF-signaling) to those promoting notochord fates in vegetally-derived A-lineages, but the order in which these factors become active is not the same in the two lineages. Experimentally-induced overlapping expression of *Foxa.a* and *Zic.r.b* in a-lineages, mimicking the situation in the notochord lineage, results in a mixed neural and notochord regulatory state in a-lineage neural precursors at early gastrula stages and notochord gene expression extending into the head at tailbud stages [84]. Thus, while the same factors are required for specification of notochord and anterior neural fate, the different temporal sequence of expression of these factors in the a-lineage cells is sufficient to promote anterior neural (‘brain’) fate and to prevent these cells from adopting axial mesoderm fate. More details on the molecular mechanisms governing these neural fate choices can be found in recent reviews [25,60,85]. 

**Figure 4:**
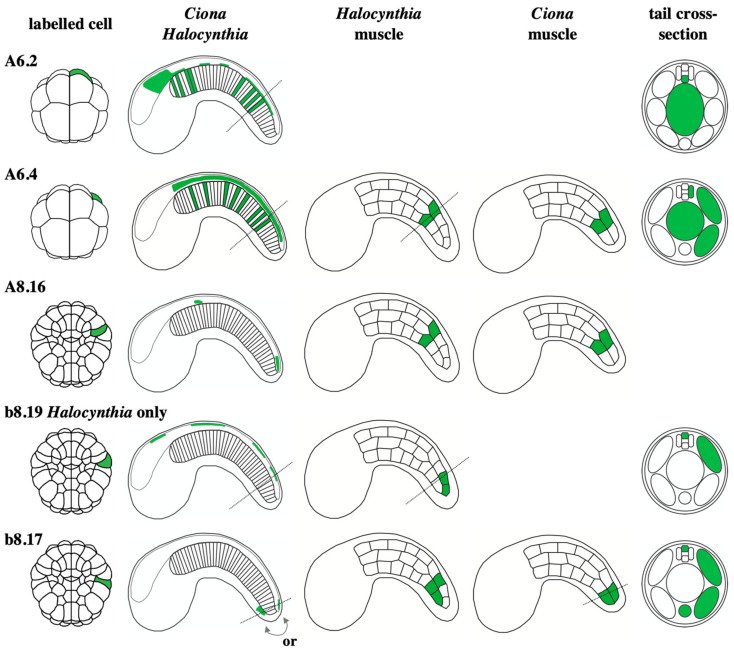
Lineage tracing of the bi-fated neuromesoderm precursors. The neuromesoderm precursor labelled is indicated on the left in green and contribution to neural and mesoderm fates indicated by green on the tailbud drawings [8,75].

The b-lineages contribute to the dorsal most row of cells in the posterior part of the CNS (Figure 3A, green cells). Although b-epidermal lineages have been shown to generate neurons of the peripheral nervous system [39,86], there are as yet no reported CNS neurons arising from these lineages. The b-neural lineage precursors arise in the lateral part of the embryo and also contribute to ectoderm, mesoderm and endoderm tissues, though there are difference between *Halocynthia* and *Ciona* (Figure 3 and Figure 4) [8]. Epidermis and neuromesodermal fates segregate at the 110-cell (early gastrula) stage of development to give two laterally-positioned neuromesodermal precursors, b8.19 and b8.17 (Figure 3B and Figure 4). In *Halocynthia* only, b8.19 cells give rise to dorsal neural tube and two muscle cells at the tip of the tail (Figure 3 and Figure 4). In *Ciona*, this cell generates only dorsal neural tube (thus following a fate choice between neural and epidermis). The b8.17 cell gives rise, in *Halocynthia*, to 3 muscle cells of the tail as well as either a dorsal neural tube cell or an endodermal strand cell (ventral row of endoderm cells at the tip of the tail), presumably one each from the left or right b8.17 cell (Figure 3 and Figure 4) [8]. In *Ciona*, each b8.17 gives rise to 2 muscle cells at the tip of the tail and either two cells of the dorsal neural tube or one cell of the dorsal neural tube and one endodermal strand cell (Figure 3 and Figure 4) [8]. This description was based on lineage-tracing. However, by following cells in embryos fixed at sequential stages of development, other authors did not report a contribution of b8.17 to the neural tube in *Ciona* [14,45]. Therefore, it remains to be confirmed whether or not b8.17 gives rise to the neural tube of *Ciona*. It is also not clear when the neural tissue segregates from muscle and/or endodermal strand in b-lineage cells.

The A-lineages will generate the lateral and ventral cells of the neural tube from the posterior part of the sensory vesicle to the tail nerve cord (Figure 3, yellow and tan cells). The A-neural lineages are closely associated with mesoderm fates, having segregated from ectoderm lineages at the 8-cell stage of development (Figure 3A). At the 32-cell stage of development, cell division segregates endoderm from the bi-fated neuromesoderm precursors, A6.2 and A6.4, positioned at the anterior margin (Figure 3B, red cells of the 32-cell stage embryo; Figure 4). These neuromesoderm precursors will generate mesoderm (mostly notochord, but also some posterior tail muscle arises from A6.4) and neural tissue (Figure 3 and Figure 4). At the 64-cell stage, A6.2 and A6.4 divide along the animal-vegetal axis segregating notochord (Figure 3B, light blue cells at the 64-cell stage) from neural/muscle fates (yellow and red cells in Figure 3B, 64-cell stage). This ‘notochord-neural’ fate segregation operates as a binary fate choice and thus these precursors can be considered as bi-fated and bi-potential [87,88]. The medial A6.2 cell generates, at the 64-cell stage, a notochord-restricted precursor and a neural-restricted precursor (A7.4, yellow) at the margin (Figure 3B). A7.4 generates the lateral and ventral posterior sensory vesicle and neck as well as the ventral row of cells in the trunk ganglion and tail nerve cord. The lateral A6.4 cells each generate, at the 64-cell stage, a notochord-restricted cell, A7.7 (Figure 3B, blue cell on 64-cell stage embryo) and a neural-muscle precursor, A7.8 (Figure 3B, red cell on 64-cell stage embryo). A7.8 subsequently divides along the medial-lateral axis to give rise to A8.15 and A8.16 (Figure 3B). A8.15 is a mono-fated neural precursor and will generate lateral cells of the trunk ganglion and tail nerve cord. A8.16 is a bi-fated neural-muscle precursor (Figure 3 and Figure 4). At neural plate stages, A8.16 divides along the anterior-posterior axis to generate one neural and one muscle precursor (Figure 3B, tan and dark blue cells at neural plate stage), via a binary fate switch (shown in *Ciona* [89]). The A-lineages are identical between the two ascidians studied, *Halocynthia* and *Ciona*, despite the observation that the molecular mechanisms specifying the muscle cells from the A-lineages has diverged [4,8,75,90,91]. The molecular mechanisms of neuro-mesoderm specification in the A-lineages will be described later (Section 6). In conclusion, a large part of the ascidian neural tube has a neuromesoderm origin.

Appendicularians (or larvaceans) belong to another order of tunicates and exhibit a tadpole body form throughout their adult lives. The cell lineages have been described and fate restriction takes place even earlier than in ascidians. Similar to ascidians, both nervous system and muscle derive from animal and vegetal hemispheres, with a large part of the CNS associated with mesendodermal (including notochord) vegetal lineages, although in appendicularians neural tissue segregates earlier from mesendoderm and there is no neuromesoderm intermediate [92,93].

## 5. Evidence of Neural-Mesoderm Lineages in Vertebrates

In vertebrates, it was believed for many years that the nervous system was induced with anterior character, following a fate choice between neural and epidermal fates. This anterior character was then ‘transformed’ by posteriorizing signals to generate the entire posterior nervous system (reviewed in [15,16]). The prevailing modern view is that only the anterior nervous system: forebrain, midbrain, hindbrain and anterior part of the spinal cord is induced and transformed from a cellular origin associated with epidermis [19,20,94,95,96]. The posterior part of the nervous system is now thought to arise predominantly from neural-mesoderm precursors (NMps) that generate the paraxial mesoderm and spinal cord [15,17,18,20,95,97,98,99,100,101]. Another neuromesodermal contribution to the vertebrate CNS is from notochord-floor plate precursors. Each of these contributions to the vertebrate CNS will be discussed in turn.

### 5.1. Neural-Somite Fate Choices

Much of the vertebrate spinal cord is believed to arise from bi-potential precursors, termed neural-mesoderm precursors (NMps), which choose between spinal cord and paraxial mesoderm (plus vasculature) fates [15,17,18,98,100,101]. Early indications for the existence of bi-potential neuromesoderm precursors came from fate mapping and mutant analysis. Fate mapping at early somite stages in mice and chick, revealed that some paraxial mesoderm and neural tissue arose from a similar location, at the node/streak border and caudal lateral epiblast and later in the caudal neural hinge of the tailbud [102,103,104,105,106]. In zebrafish, single-cell fate mapping identified bi-fated neural-muscle precursors at the late blastula stage, which segregate into mono-fated precursors by the early gastrula stage [20,107]. 

Analysis of mutant cells in mice also indicated a close relationship and potential bi-potentiality between paraxial mesoderm and spinal cord. Examples include *FgfR1*-mutant cells failing to pass through the primitive streak to form mesoderm and instead forming secondary neural tubes [108]; *Wnt-3a* mutant cells that ingress through the primitive streak, but fail to migrate laterally to form somites, instead giving rise to secondary neural tubes [109]; and *Tbx6* mutants in which posterior somites are converted into supernumerary neural tubes [110]. The interactions between these and other factors and how they drive paraxial mesoderm versus spinal cord fate are now well understood and appear to be conserved among vertebrates [15,17,18,97,98,111,112,113,114,115]. 

Clonal analysis in mice proved unequivocally that bi-fated neuromesoderm precursors contribute to the neural tube [96]. In mice, while segregation of neural from ectoderm takes place early in development, self-renewing neuromesoderm precursors (neural-somite) continuously contribute to the posterior nervous system (spinal cord) during body axis elongation [96]. Retrospective clonal analysis in zebrafish also revealed that mid- and posterior spinal cord has a closer clonal relationship to muscles derived from paraxial mesoderm than it does to anterior nervous system, strongly arguing for a neural-mesoderm fate choice at the base of the generation of a large part of the spinal cord [20]. Unlike in mice in which neuromesoderm precursors exhibit self-renewing properties during the generation of the spinal cord, in zebrafish, much of the segregation of neural from paraxial mesoderm fates appears to take place earlier, during early gastrulation [20,107]. Resultant mono-fated pools of spinal cord or somite precursors nonetheless retain the capacity to change developmental status between neural and mesoderm, depending on Wnt signaling in the tailbud [100]. Thus, at least in the tailbud, these cells can be considered to remain bi-potential. A small population of NMps retained in the tailbud are largely quiescent during somitogenesis stages and are thought to contribute to only last region of the body axis [20,100,116]. 

Bi-potential NMps have been successfully created from mouse embryonic stem cells by recreating embryonic conditions (Wnt plus FGF signaling), demonstrating that precursors of posterior spinal cord and paraxial mesoderm pass through a bi-potential state [97]. Anterior neural tissue up to hindbrain levels follows a distinct transcriptional trajectory compared to NMps that generate spinal cord, further confirming the different origins of anterior and posterior nervous system [97]. Furthermore, evidence suggests that anterior and posterior regional identity is imposed on cells prior to their acquisition of neural identity [95]. Specific anterior and posterior enhancers of the pan-neural *Sox2* gene in chick and mice provided additional evidence for distinct mechanisms controlling anterior and posterior CNS development [18]. 

Thus, in vertebrates, at least the posterior spinal cord is generated with a significant contribution from cells that choose between neural and mesoderm (somite) fates [15,18,20,95,101]. The precise extent of the NMp contribution along the anterior-posterior axis of the neural tube is not yet clear, as discussed by Henrique et al., [15].

### 5.2. Neural-Notochord Fate Choices

There is also evidence for another type of neuromesoderm precursor, which generates the ventral-most part of the neural tube, in particular, the medial floor plate, and notochord in vertebrates. The vertebrate floor plate consists of medial and lateral cell types, which share the expression of a subset of genes [117,118,119,120]. It is the medial floor plate that is associated with notochord fates.

Fate mapping in mice [121,122], chick [105,117] and zebrafish [123,124,125,126,127,128,129] show that floor plate precursors reside in the organizer (early gastrula organizer, node, shield), in a similar region to notochord precursors and in a distinct location compared to those of the rest of the spinal cord. 

In chick, bi-fated notochord-floor plate precursors have been identified in the node by single cell labelling [130]. In zebrafish, bi-fated notochord-ventral neural precursors were also found at blastula stages although it is unlikely that these cells are committed in any way [21,128,131]. By the early gastrula stage in zebrafish, notochord and floor plate precursors have segregated into distinct regions of the shield [107,127,128,131,132]. Specification of floor plate identity begins early, during gastrulation, with floor plate character actively maintained in the tailbud at later stages [123,133,134,135]. In mice, fate segregated (i.e., mono-fated) notochord and floor plate precursor pools occupy different regions of the node [122,136,137]. This is consistent with the observation that retrospective lineage tracing in mice identified only one clone contributing to notochord and neural cells (as well as posterior paraxial mesoderm) [96] and suggest that notochord-neural precursors are either very rare or segregate early during development and do not exist as a bi-fated precursor pool.

Evidence for bi-potential notochord-neural precursors can be found under certain conditions in mice, chick and zebrafish. In both chick and zebrafish, mutations or manipulation of signaling pathways result in fate switches between notochord and floor plate fates revealing the presence of bi-potential floor plate-notochord precursors under these conditions [107,132,133,138,139,140]. For example, several mutations effecting notochord formation in zebrafish result in larger medial floor plates in the hindbrain and spinal cord [118,120,123,132,139,140,141,142]. Strikingly, while wild type blastula cells transplanted into the dorsal margin cells of a wild type host generate predominantly notochord, transplantation of *ntl* (*brachyury*, a key notochord determinant) mutant cells under the same conditions results in a switch from a notochord to a floor plate contribution [132]. Similarly, in chick, manipulation of the Notch signaling pathway results in notochord-floor plate fate transformations at the level of the spinal cord and posterior hindbrain [138]. In mouse early somite stage embryos (E8.5), transplantation of the rostral node, which gives rise exclusively to notochord, into the border of the primitive streak (that usually produces ventral neural tube and paraxial mesoderm) converts the rostral node notochord precursors into a ventral neural tube fate (not restricted to floor plate) [104]. Transplantation of rostral node cells into the anterior primitive streak, which normally generates paraxial mesoderm and tail bud mesoderm, also converted the rostral node cells from notochord to neural fates. Thus, the rostral node cells can switch from notochord to neural fate even if they do not generate neural fates under normal developmental conditions [104]. 

Along the anterior-posterior axis, the floor plate cells appear to arise from distinct origins and by distinct mechanisms [119]. In mice, the ventral midline of the fore and midbrain is associated with organizer tissue that arises prior to node formation (early and mid-gastrula organizers, which also generates head mesendoderm) [121] and *Shh* (*Sonic hedgehog*) expression in anterior and posterior floor plate are controlled by distinct enhancers [143]. In chick embryos, anterior floor plate (from hindbrain levels) is induced by prechordal mesoderm in a region of epiblast just anterior to the node called ‘area a’; a distinct population of cells compared to more posterior floor plate precursors that arise together with notochord within the node itself [144]. The allocation of cells to anterior and posterior floor plate does not appear to be absolute and is not yet fully understood [119]. 

In zebrafish, anterior and posterior floor plate expression of *Shh* is also driven by distinct enhancers [120] and mutations differentially affect either the entire floor plate or specifically the posterior floor plate, suggesting mechanistic differences along the anterior-posterior axis [120,124,132,139]. For example, mutations of the notochord-promoting T-box factor, *ntl* (*Brachyury*), are thought to result in conversion of notochord precursors to a floor plate fate [132,139]. *Ntl* mutations can rescue posterior medial floor plate (MFP) up to the hindbrain, in a *Nodal* (*cyc*) mutant background, which would otherwise have no MFP along the entire anterior-posterior axis [139]. Thus, expression of *ntl* can govern the bi-potentiality of notochord precursors to choose between notochord and floor plate fate, at least in the posterior neural tube of zebrafish. We can conclude that bi-fated and bi-potential notochord-neural (floor plate) cells exist in vertebrate embryos. Exactly when these different fates become committed and the exact extent of their anterior-posterior contribution is not entirely resolved [119,120]. Nonetheless, from the available evidence we can conclude that vertebrate notochord-floor plate precursors contribute to at least the posterior part of the neural tube.

### 5.3. A Note on the Historical Controversy Surrounding Floor Plate Induction in Chick

There has been some controversy surrounding the formation of the floor plate in chick. Whether floor plate cells are generated as a specific precursor and laid down in the ventral neural tube or whether floor plate is induced in the ventral neural tube has been the subject of much debate [145,146]. Nonetheless it seems now to be accepted that, at least the developmental origin of medial floor plate cells is distinct from those of the rest of the neural tube, and is closely associated with axial mesoderm [120,145,146]. The main contention appears to be whether the cells that share a common origin with notochord are laid down and then induced to form floorplate or whether they are laid down already with floor plate character [120,145,146]. Part of this confusion is likely to have stemmed from the plasticity within the neural tube to form floor plate (e.g., [117,138]) and the fact that there are two types of floor plate, medial and lateral, of which only the medial cells share a common embryonic origin with axial mesoderm [117,118,120].

## 6. Molecular Mechanisms of Neuromesoderm Fate Segregation in Ascidians

The molecular mechanisms of neural fate segregation are well described in ascidians, particularly in a- and A-lineage [25,60,85]. Despite the similar final body plans shared between ascidian and vertebrate larvae, they often do not appear to develop using the same molecular strategies [90]. Even among ascidian species, the molecular mechanisms required to determine equivalent cell types in equivalent positions can be remarkably divergent [4,5,7,10,91,147,148,149]. In the following discussion, where differences are found between *Ciona* and *Halocynthia*, the *Ciona* data is described. The *C. robusta* unique gene model IDs for genes discussed in this section are provided at the end of the review (Section 9).

**Figure 5:**
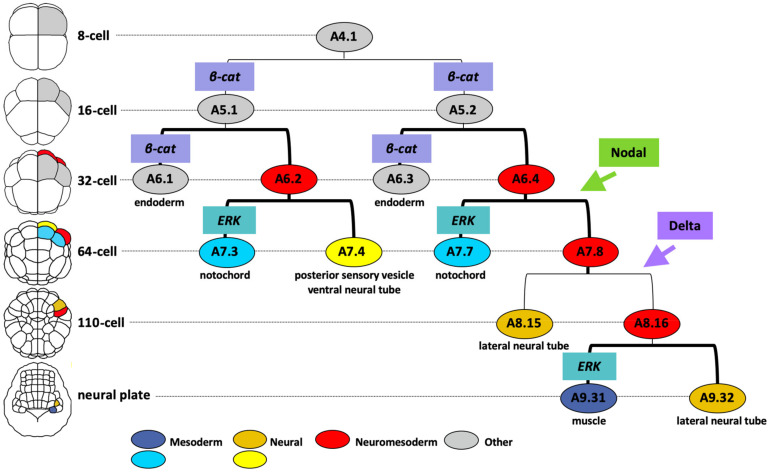
Molecular mechanisms of A-lineage neuromesoderm segregation in *Ciona* from 8-cell stage to neural segregation. One A4.1 founder lineage is shown (the other side is identical). Cells are colored following the key at the bottom. β-catenin and ERK activity is depicted by boxes on the lineage bars, just above the cell name; signals arising from neighboring cells (Nodal and Delta) are depicted by boxes and arrows. Thick lineage brackets indicate cell division along the animal-vegetal axis (16-to-32-cell stage, 32-to-64-cell stage) or anterior-posterior axis (neural plate stage), whereas thin brackets indicate cell division along the medial-lateral axis. The positions of cells following each cell division is shown on the embryo drawings on the left, using the same color code, with the stages indicated.

The main neuromesodermal lineage is the A-lineage, arising from the A4.1 blastomere pair at the 8-cell stage of development. A brief overview of the molecular mechanisms leading to neural segregation from the A-lineage is shown in Figure 5. Between the 8- and 16- cell stage β-catenin/TCF transcriptional activities are detected in the vegetal cells, including A5.1 and A5.2 of the A-lineage [150]. β-catenin is required in A5.1 and A5.2 for the specification of endoderm and margin (mesoderm/neural) cell identity and for repression of ectoderm fates [147,150,151,152]. The upstream event leading to nuclear translocation of β-catenin is unknown. At the 32-cell stage, differential activation of β-catenin promotes endoderm over notochord-neural (red cells in Figure 5) fates during this binary fate choice [147,152]. The subsequent binary fate choice sees the notochord lineage (blue cells in Figure 5) segregating from the neural lineage (plus some muscle) at the 64-cell stage with ERK activity in the notochord precursors promoting notochord over neural fates [87,88,153]. The differential pattern of ERK activity is driven by FGF9/16/20 (promoting the Ras-ERK cascade) and the ephrin ligand Efna.d (attenuating the cascade) [88,153,154]. Thus, the segregation of A-neural (plus some muscle)-notochord lineages from the 16- to 64-cell stages requires the following sequence of signaling inputs: β-catenin-ON, β-catenin-OFF and then ERK-OFF (for neural, plus some muscle) or ERK-ON (for notochord). Interestingly, in the vegetal hemisphere of the embryo, the arrangement of notochord and neural precursors in the A-lineage cells is mirrored by a similar arrangement of mesenchyme and muscle precursors in the B-lineage cells of the vegetal hemisphere and these fate segregations are both driven by FGF/ERK signaling with FGF required for notochord and mesenchyme fates that segregate from notochord-neural and muscle-mesenchyme precursors respectively [155]. The attribution of notochord/neural fates to the A-lineages and muscle/mesenchyme fates to the B-lineages is itself governed by the inheritance of a maternal determinant *macho-1* (later renamed *Zic.r.a*) to the B-lineages [155]. Removal of *macho-1* results in muscle lineages adopting neural fates and mesenchyme lineages adopting notochord fates [155].

Across the medial-lateral axis of the embryo, Nodal- and Delta-like- signals arising from bilaterally-positioned neighboring cells are required to specify the lateral-most cells of the neural lineage from the 32-cell stage and 64-cell stage, respectively (Figure 5) [26,89,156,157,158]. In the absence of Nodal the A7.8 neuromesoderm precursor adopts fates consistent with an A7.4 identity, whereas the absence of Delta-like leads to a loss of A8.16 lineage identity [4,26,89,156]. The final lineage segregation between neural and mesoderm takes place in this lateral cell at the neural plate stage (mid-gastrula) when differential ERK activity specifies mesoderm (muscle) over neural fate (Figure 5) [89]. This final neuromesoderm segregation thus follows the sequence, from the 16-cell to neural plate stage, of β-catenin-ON, β-catenin-OFF, Nodal-ON, ERK-OFF, Delta/Notch-ON and finally ERK-OFF (for neural fate) or ERK-ON (for muscle fate).

The accumulation of β-catenin in the vegetal lineages of early ascidian embryos is an evolutionary conserved mechanism among metazoans for establishing the primary body axis, with β-catenin defining the site where mesendoderm forms and gastrulation begins [159,160]. In ascidians, the A-lineage neuromesoderm precursors arise in this vegetal embryonic domain and require a β-catenin ON to OFF sequence of activation for the initiation of their specification [147]. The a- and b-lineage derived CNS arises in the animal, or β-catenin OFF, embryonic hemisphere.

In ascidians, the specification of the ventral neural tube appears very different compared to floor plate specification in vertebrates, which requires Nodal and SHH (sonic hedgehog) signaling [119,120]. It seems unlikely that Hedgehog plays a similar role in ascidians. Although a *hedgehog* gene (*hh-2*) is expressed in the cells of the ventral neural tube (but notably not in the notochord), this expression is late, from tailbud stages, and there is no evidence that it plays a patterning role in the neural tube of ascidians as it does in vertebrates [161,162,163,164,165]. In ascidians, Nodal is also not implicated in notochord-neural specification, but rather patterns the neural plate to specify lateral over medial identities and actually represses the ventral neural and posterior sensory vesicle fate, restricting their formation to the medial neural lineages [156,166,167]. This is in stark contrast to the positive role for Nodal in floor plate development in vertebrates [120].

In vertebrates, an interplay between Wnt-, FGF- and Retinoic acid (RA-) signaling pathways act to both maintain neuromesoderm (neural-somite) progenitors (*Brachyury* (*T*)*+*/*Sox2+*) and promote their transition to neural (RA) or mesoderm (Wnt, FGF) via promotion of *Brachyury* (*T*) and *Tbx6* in the mesoderm and *Sox 2* in the neural precursors [115]. The *Ciona Sox 1/2/3* gene is not specifically expressed in neural tissue at early stages in ascidians [168]. A role for Wnt- and RA- signaling in A-lineage neuromesoderm formation has not been described in *Ciona*. In *Halocynthia*, the mechanisms of A-lineage muscle specification involve similar cellular interactions to those observed in *Ciona*, but employ different signaling molecules. In *Halocynthia*, Wnt-5 is required for formation of muscle cells from both the neuro-muscle A- and b-lineages, although, at least for the A-lineage, this cellular interaction takes place before gastrulation [4,91].

Some parallels can be drawn from comparisons of FGF-signaling between ascidians and vertebrates. FGF-signals are transiently required for onset of activation of the pan notochord-neural gene *Zic-r.b* in the neuromesoderm precursors at the 32-cell stage [169]. At each neuromesoderm lineage segregation, FGF/ERK promotes mesoderm over neural fates, similar to some situations in vertebrates, and activates *Brachyury* and *Tbx6* in segregated notochord and A-lineage muscle precursors respectively [88,89,108,115,153,170].

In vertebrates, expression of the posterior promoting transcription factor *Cdx* is downstream of Wnt [17,171]. However, in ascidians, a combination of ERK activity, Nodal and Delta are required for *Cdx* expression in the A-lineage derived neural plate at the mid-gastrula stage, in the lateral, posterior-most row of cells, including the muscle precursor after it has segregated from neural lineages [26,89,157]. These cells will give rise to part of the ventral trunk ganglion and tail nerve cord, the lateral tail nerve cord and the A-lineage tail muscle [8,13]. At tailbud stages *Cdx* is required in the anterior part of the tail nerve cord to repress more anterior gene expression, but does not appear to be upstream of *HOX* gene expression as is the case in the vertebrate spinal cord [17,26,171]. Its role in A-lineage muscle specification has not been addressed.

Interestingly, despite the absence of self-renewing precursors or so-called ‘posterior growth’ in ascidian tailbuds, both Wnt (*Wnt5* and *Wnttun5* (*tunicate Wnt family member 5*)) and FGF (*FGF8/17/18* and *FGF9/16/20*) ligands are expressed in the tail-tip precursors of *Ciona* from mid-gastrula stages and retinoic acid (RA) synthesizing enzyme (*Raldh2*) is expressed more anteriorly in the anterior tail muscle [26,154,168,171,172]. In *Ciona*, RA is required for *HOX-1* gene activation in the neck, trunk ganglion and anterior tail nerve cord as well as the adjacent epidermis at tailbud stage and Wnt signals are required for *HOX-12* expression in the posterior most CNS [26,173,174,175]. However, the *HOX* genes play only a limited role in ascidian larval CNS development [176]. Wnt-, FGF- and RA- signals are involved in patterning the epidermis and associated peripheral nervous system of the ascidian larvae and FGF8/17/18 and Wnt-5 ligands are required for correct tail tip cell morphology and tail elongation [172,175,176]. Thus, while it seems that the posterior patterning mechanisms that operate in vertebrates are functioning in ascidians, they do not appear to play a major role in posterior CNS development or NMp specification in the A-lineage. It would be extremely interesting to address if these patterning mechanisms are involved in the fate choice between neural and mesoderm in the neuromesoderm precursors of the b-lineage, which is found at the very tip of the tail and about which very little is known.

We conclude that the mechanisms of neuromesoderm specification in ascidians and vertebrates have, in the most part, diverged significantly.

## 7. A Common Origin for Neuromesoderm Precursors at the Base of Chordates?

**Figure 6:**
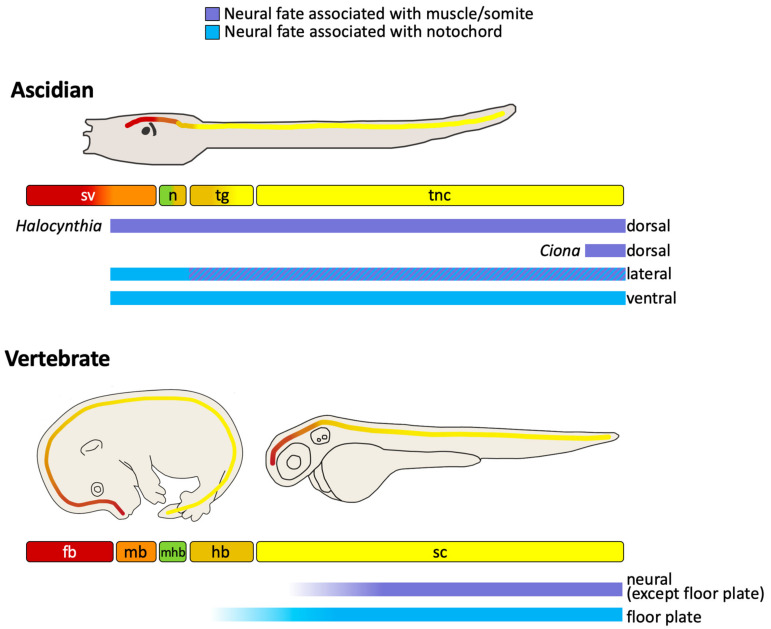
Relative contribution of neuromesoderm precursors to the larval CNS in ascidians and vertebrates along the anterior-posterior axis. The animal drawings show the positions of the equivalent CNS regions along the anterior-posterior axis, with the same color code as in Figure 2. MHB is not colored. Mouse and zebrafish drawings are taken from ref.[101](part of their Figure 3), with permission from Elsevier. Below the drawings is a schematic representation of the anatomical compartments with colors showing equivalent regions, from Figure 2. For ascidians, model iv is taken. Below the models the contributions of neuromesoderm (bi-fated or bi-potential) precursors is indicated, with neural-somite/muscle precursors in purple and neural-notochord in blue. The diagonal striped colored box represents the A7.8 lineage, which segregates from notochord at the 64-cell stage as a neural-muscle precursor. Ascidian neuromesoderm contributions extent into more anterior regions of the CNS compared to vertebrates. Vertebrate summary based on [20,119,120,144]. sv, sensory vesicle; n, neck; tg, trunk ganglion; tnc, tail nerve cord; fb, forebrain; mb, midbrain; mhb, mid-hindbrain boundary; hb, hindbrain; sc, spinal cord.

In summary, neuromesodermal precursors make extensive contributions to both the ascidian and vertebrate CNS (Figure 6). In ascidians, the anterior part of the CNS, the anterior sensory vesicle, is generated from the a-lineages, following a cell fate choice between neural and epidermis. Much of the remaining CNS, which constitutes a large part, is generated from neuromesodermal precursors originating from the vegetal hemisphere (Figure 3, Figure 4 and Figure 6). These lineages contribute to the lateral and ventral neural tube from the posterior part of the sensory vesicle to the tail nerve cord following a cell fate choice between neural and notochord, with a small part arising from the A8.16 lineage that undergoes an additional lineage segregation between neural and muscle (Figure 3 and Figure 4). Small parts of the dorsal neural tube are generated from the b8.17 lineage, which also gives rise to muscle and endodermal strand cells at the tip of the tail, although its contribution to the neural tube requires confirmation in *Ciona*. The b8.19 lineage generates most of the dorsal neural tube in both *Ciona* and *Halocynthia*. While this lineage also generates the tail-tip muscles in *Halocynthia*, it is neural-restricted in *Ciona* (Figure 3 and Figure 4).

Neuromesoderm-derived neural precursors also contribute to the generation of the vertebrate CNS. Cells that choose between notochord and neural fates generate the floor plate at least at posterior levels, whereas cells that choose between somite and neural fates give rise to a large part of the spinal cord (Figure 6). Thus, in both ascidians and vertebrates, neuromesoderm precursors are contributing to posterior parts of the CNS.

In ascidians, distinct molecular mechanisms are involved in specification of neural fates from the different lineages of the CNS. For example, FGF is required for ‘neural induction’ in a- and b-lineages [76], whereas inhibition of FGF-signaling is required for neural specification in A-lineages (Figure 5; 64-cell stage) [88]. This is consistent with the hypothesis proposed in vertebrates for distinct molecular mechanisms driving the formation of the anterior and posterior CNS [15,17,18,19,177].

### 7.1. Neural-Muscle Precursors

While a large part of the vertebrate spinal cord shares a common origin with somite as discussed above, the contribution of bi-fated neural-muscle precursors to the ascidian neural tube is clearly more limited; just a few cells, mostly at the tip of the tail, originate from the lateral A8.16 and b8.17 cells of the early gastrula stage embryo, although the b8.19 neuromesoderm precursor contributes more extensively to the dorsal neural tube in *Halocynthia* (Figure 3, Figure 4 and Figure 6). While the neuromesodermal lineages are associated with self-renewal at certain axial levels in mice [96], this is limited in zebrafish [20,100] and not at all the case in ascidians.

It would be very interesting to address the origin of muscles in much larger ascidian larvae in which a process known as “caudalization” takes place. Caudalization describes the addition of muscle cells to the tadpole tail without changing the number of cells in other tissue types (i.e., the notochord remains at 40 cells) [178]. These larvae, which some colonial ascidians produce, can contain more than 1000 muscle cells, compared to the 42 of *Halocynthia* or 36 in *Ciona* [9,178,179]. These additional muscle cells arise by proliferation [180]. It would be extremely interesting to determine if, in these colonial species with giant larvae, the additional muscles of the tail originate from the neural-mesoderm lineages. Interestingly, in *Halocynthia*, which exhibits a minimal “caudalization” with the addition of six muscle cells to the tail, the additional muscle cells arise from b-lineage neural-muscle precursors (Figure 3 and Figure 4) [8,178].

Like in vertebrates, the ascidian neural-muscle precursors are associated with posterior neural tube, and germ layer segregation takes place late during development; the segregation of neural and muscle lineages in A- and b-lineages represents one of the latest events of germ-layer segregation to take place [8] However, unlike in vertebrates, A-lineage neural-muscle precursors do not persist beyond gastrulation with their lineage segregation taking place within the neural plate at the mid-gastrula stage (Figure 3B) [96,100,101]. It has not been reported when muscle and neural fates segregate in the b-lineage. The presence of neural-muscle (or somite) precursors in both vertebrates and ascidians supports their presence in the last common ancestor of Olfactores (here and [9]) but the extent of their contribution to the neural tube is variable.

### 7.2. Notochord-Neural Precursors

Neural tissue arising from bi-fated and bi-potential notochord-neural precursors generate a large part of the CNS of ascidians (Figure 3, Figure 4 and Figure 6). The A6.2 cell of the 32-cell stage embryo is a bi-fated notochord-neural precursor that contributes cells up to forebrain levels in the CNS. The ‘forebrain’ contribution of A-lineage cells is based on the A-lineage origin of the ocellus-associated ciliary photoreceptor cells, most likely corresponding to forebrain-derived retinal or pineal photoreceptors of vertebrates [36,38,53]. At the level of the trunk ganglion and tail nerve cord, A6.2 also contributes to the ventral row of cells (Figure 3 and Figure 4). This ventral contribution bears a strong resemblance to the notochord-neural precursors that generate the medial floor plate of vertebrates. In both ascidians and vertebrates, *FoxA* and *Hedgehog* genes are expressed in these ventral cells, although so far there is no evidence that either the notochord or floor plate plays a role in patterning the neural tube in ascidians as they do in vertebrates [17,67,119,162,165,181,182]. The other ‘notochord-neural’ precursors of the 32-cell stage embryo, the A6.4 cell, give rise to notochord, a few posterior muscle cells and to all the lateral cells of the trunk ganglion and tail nerve cord (‘hindbrain-spinal cord’) (Figure 2, Figure 3 and Figure 4). Together, the ascidian notochord-neural precursors generate a much larger part of the neural tube compared to vertebrates; all ventral and lateral cells from ‘posterior forebrain’ (photoreceptors) to ‘spinal cord’ (Figure 6). Despite these differences in the levels of CNS contribution, a common origin for neural and notochord shared between ascidians and vertebrates is nonetheless striking and argues once more for common neuromesodermal origin of part of the neural tube at the base of chordates. Interestingly, the segregation of notochord from neural takes place very early in development in ascidians, prior to gastrulation, similar to zebrafish and perhaps also mouse, although it has not yet been demonstrated if notochord-floor plate cells arise from an early common precursor in mouse.

### 7.3. Ascidian b-Lineage Cells: Neural, Muscle, Endoderm

The contribution of b8.17 cells to dorsal neural tube, muscle and endodermal strand in the tail tip bears some resemblance to axial precursors in the tailbud of zebrafish and chick which generate floor plate, notochord and hypochord (probable endoderm underlying the notochord of zebrafish in a similar position to the endodermal strand of ascidians) [100,105,126,130,133,182,183]. However, the ascidian b-lineage cells contribute to dorsal rather than ventral neural tube and muscle rather than notochord. In addition, these precursors arise at the lateral border of the future neural plate of ascidian embryos, a region proposed to be an evolutionary precursor of neural crest/placode [30,39,56,79]. A proto-neural crest character may explain why b-lineage cells contribute to multiple germ layers (epidermis, neural tube, muscle, endoderm) and may account for the relatively late tissue fate restriction of cells that originate in these lineages [8]. On the other hand, the b8.17 lineage may also represent tailbud-like neural-muscle-endoderm precursors, with the b8.19 lineage of *Halocynthia* representing a tailbud-like muscle-neural precursor (NMp). Understanding more about the lineage segregation of b-lineage cells and their specification will help shed light on whether they are more neural-border-like or tailbud precursor-like (axial or NMp).

## 8. Conclusions

We conclude that, in both vertebrates and ascidians, part of the posterior neural tissue shares a common origin with mesoderm, although the mechanisms of specification differ. We therefore propose that a shared developmental origin of mesoderm and neural tissue in ascidian embryos is not a peculiarity of ascidians but descended from a common ancestor in which some neural tissue originates from a neural-epidermis fate choice and some from a neural-mesoderm fate choice and that the extent of these contributions has come to vary in different evolutionary lineages.

While a different embryonic origin of the anterior and posterior CNS in ascidians is entirely consistent with the proposed independent origin of anterior and posterior CNS in vertebrates [15,17,18], the extent of these differential contributions is not the same between ascidians and vertebrates. To explain how the boundary between neuro-epidermis and neuro-mesoderm contributions to the CNS does not appear to be fixed in different chordate embryos, we suggest that the mechanisms that pattern the CNS along the anterior-posterior axis and those that establish neural identity may act, and therefore evolve, independently (or can at least be uncoupled), as suggested by recent work in mice [95].

It has been experimentally demonstrated that cells following distinct transcriptional trajectories can converge upon a similar cell type [184] (see also [92,185]). This suggests that cells from distinct embryonic lineage origins, undergoing distinct transcriptional trajectories should be able to seamlessly unite to form a single functional unit, in this case the CNS. However, this is not unique to the CNS; for example, in ascidians, in addition to CNS, both notochord and muscle arise in distinct lineages via distinct molecular mechanisms [4,9,85]. Ascidians could provide an excellent model system to address the process whereby distinct embryonic and transcriptional trajectories ultimately converge upon a similar cell type.

## 9. *C. robusta* Unique Gene IDs

From the KH2012 *C. robusta* genome assembly. See Ghost (http://ghost.zool.kyoto-u.ac.jp/otherfr_kh.html, accessed 7 April 2021) ([168,186] and ANISEED (http://www.aniseed.cnrs.fr/, accessed 7 April 2021) [187] for further information. *Wnt 5* (KH.L152.45), *Wnttun5 (tunicate Wnt family member 5*, KH.C9.257), *FGF9/16/20* (KH.C2.125), *FGF8/17/18* (KH.C5.5), *ERK* (KH.L153.20), *Nodal* (KH.L106.16), *Delta-2/Delta-like* (KH.L50.6), *β-catenin* (KH.C9.53), *Hedgehog-2* (KH.C5.544), *Raldh2* (KH.C4.697), *Sox2/14/21* (KH.S164.12; KH.C1.99), *Cdx* (KH.C14.408), *Brachyury/T* (KH.S1404.1), *Snail* (KH.C3.751), *Tbx6* (a multi-copy gene [188], KH.S654.3- *tbx6b*); *HOX-1* (KH.L171.16), *HOX-12* (KH.C7.472), *Zic.r.b* (a multi-copy gene [189], KH.S816.1), *macho*/*Zic-r.a* (KH.C1.727), *Gata.a* (KH.L20.1).

## Data Availability

No new data is reported in this study. Links to web sites accessed are found in Section 9.
